# Brucellosis infection complicated with myelitis: a case report and literature review

**DOI:** 10.3389/fcimb.2024.1378331

**Published:** 2024-05-16

**Authors:** Xiaoyu Ma, Ying Wang, Qiong Wu, Xiaomei Ma, Qiang Wang, Qinghong Guo

**Affiliations:** ^1^ The First Clinical Medical College, Lanzhou University, Lanzhou, China; ^2^ Department of Gastroenterology, The First Hospital of Lanzhou University, Lanzhou, China; ^3^ Gansu Province Clinical Research Center for Digestive Diseases, The First Hospital of Lanzhou University, Lanzhou, China; ^4^ Department of Neurology, The First Hospital of Lanzhou University, Lanzhou, Gansu, China; ^5^ School of Medicine, Qinghai University, Xining, Qinghai, China

**Keywords:** neurobrucellosis, myelitis, MetaCAP™, ganglioside, hormone therapy

## Abstract

Brucellosis is a zoonotic disease caused by a Gram-negative coccus a facultative intracellular pathogen. Neurobrucellosis has an incidence rate of 3-7% among all patients with brucellosis, while spinal cord involvement is rare and carries a significant mortality risk. This report describes a case of brucellosis myelitis in a 55-year-old male patient who presented with recurrent paralysis, incontinence, and damage to the visual and auditory nerves. The diagnosis of neurobrucellosis involves a serum tube agglutination test, cerebrospinal fluid analysis, a physical examination of the nervous system, and a comprehensive review of the patient’s medical history. The presence of brucellosis was confirmed in cerebrospinal fluid using MetaCAP™ sequencing. Treatment with a combination of rifampicin, doxycycline, ceftriaxone sodium, amikacin, compound brain peptide ganglioside, and dexamethasone resulted in significant improvement of the patient’s clinical symptoms and a decrease in the brucellosis sequence count in cerebrospinal fluid. For the first time, MetaCAP™ sequencing has been used to treat pathogenic microbial nucleic acids, which could be a valuable tool for early diagnosis and treatment of neurobrucellosis.

## 1 Introduction

Brucellosis is a zoonotic infectious disease caused by bacteria of the genus Brucella. Approximately 500,000 new cases of human brucellosis are reported worldwide each year, posing a substantial threat to the social economy and the physical and mental health of humans (Shi et al., 2023). It is caused by aerobic, dynamic, gram-negative facultative intracellular bacteria of the Brucella genus, with an incubation period typically ranging from 2 to 4 weeks (Shakir, 2021). Humans become infected through the consumption of raw dairy products and undercooked meat or by directly contacting animals, their placentas, or aborted fetuses that are infected (D'Anastasio et al., 2011). This disease exhibits prominent occupational characteristics, with rural workers and butchers being particularly susceptible ( (Shi et al., 2023). Neurobrucellosis (NB) refers to the involvement of the nervous system and occurs in 3-7% of all patients with brucellosis (Haji-Abdolbagi et al., 2008). The most frequently observed manifestations of neurobrucellosis include meningitis and meningoencephalitis, myelitis, craniocerebral diseases, peripheral neuropathy, and others (Soares et al., 2023). NB has a slow onset., The effects of brucellosis on the nervous system may result from persistent intracellular microorganisms (Dando et al., 2014), or infection may trigger immune mechanisms, resulting in inflammatory immune responses of cytokines or endotoxins and leading to myelopathy and/or demyelination inflammatory central and peripheral demyelination (Ceran et al., 2011). NB belongs to the acute and critical type of brucellosis, but due to the lack of specific clinical manifestations, there is a high rate of early misdiagnosis, If left untreated in time, it may lead to death or permanent complications, which requires a high level of suspicion (Moosazadeh et al., 2016). Therefore, patients with nervous system symptoms in high epidemic areas should always be considered for NB (Dreshaj et al., 2016).In this report, we present a unique case of recurrent brucellosis myelitis, which represents the first instance of diagnosing brucellosis myelitis using high-throughput nucleic acid sequencing of MetaCAP™ pathogenic microorganisms.

## 2 Case report

**Figure 1:**
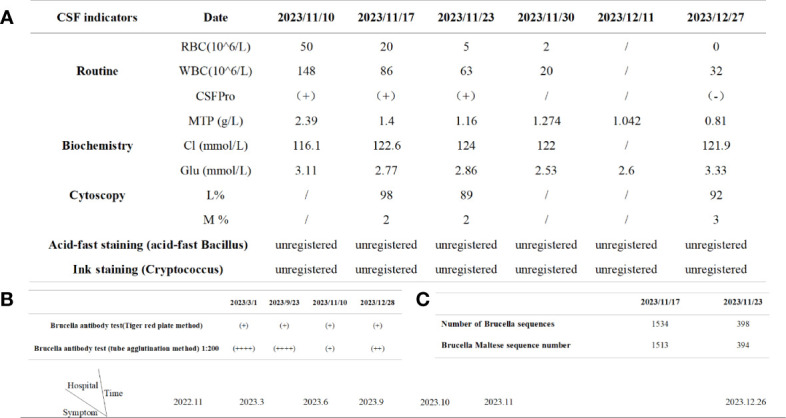
Timeline of the patient's relevant tests. **(A)** Cerebrospinal fluid results during the course of the disease; **(B)** Results of brucella antibody detection during the course of disease; **(C)** Metacap sequencing.

**Figure 2:**
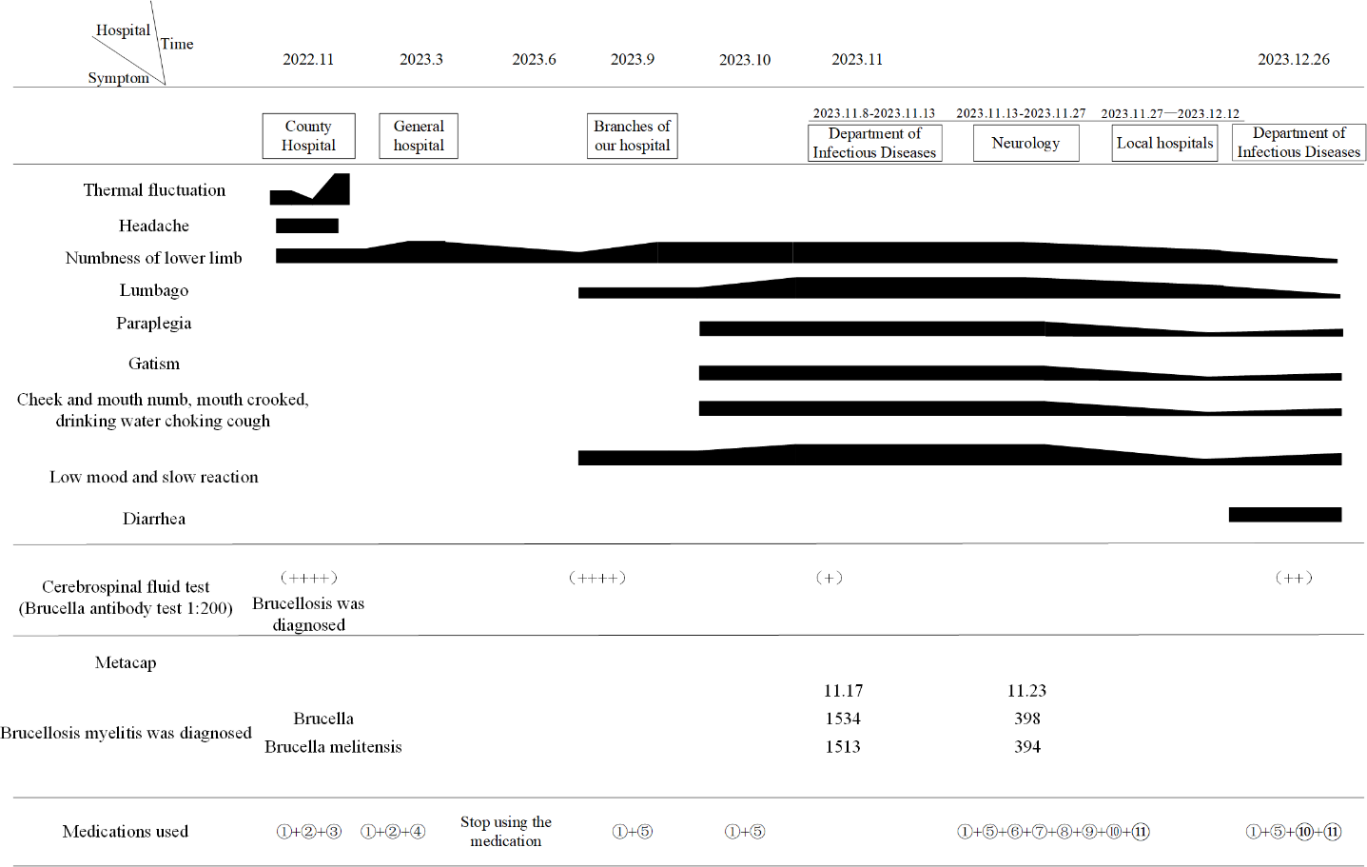
Disease progression chart. Therapeutic: ①Doxycycline; ②Cefixime dispersible tablet; ③Ciprofloxacin ; ④Moxifloxacin; ⑤Rifampicin; ⑥Amikacin; ⑦Ceftriaxone sodium for injection; ⑧Dexamethasone sodium phosphate injection; ⑨Compound brain peptide ganglianzhi injection; ⑩Mecobalamine; ⑪Vitamin B1 tablet.

**Figure 3:**
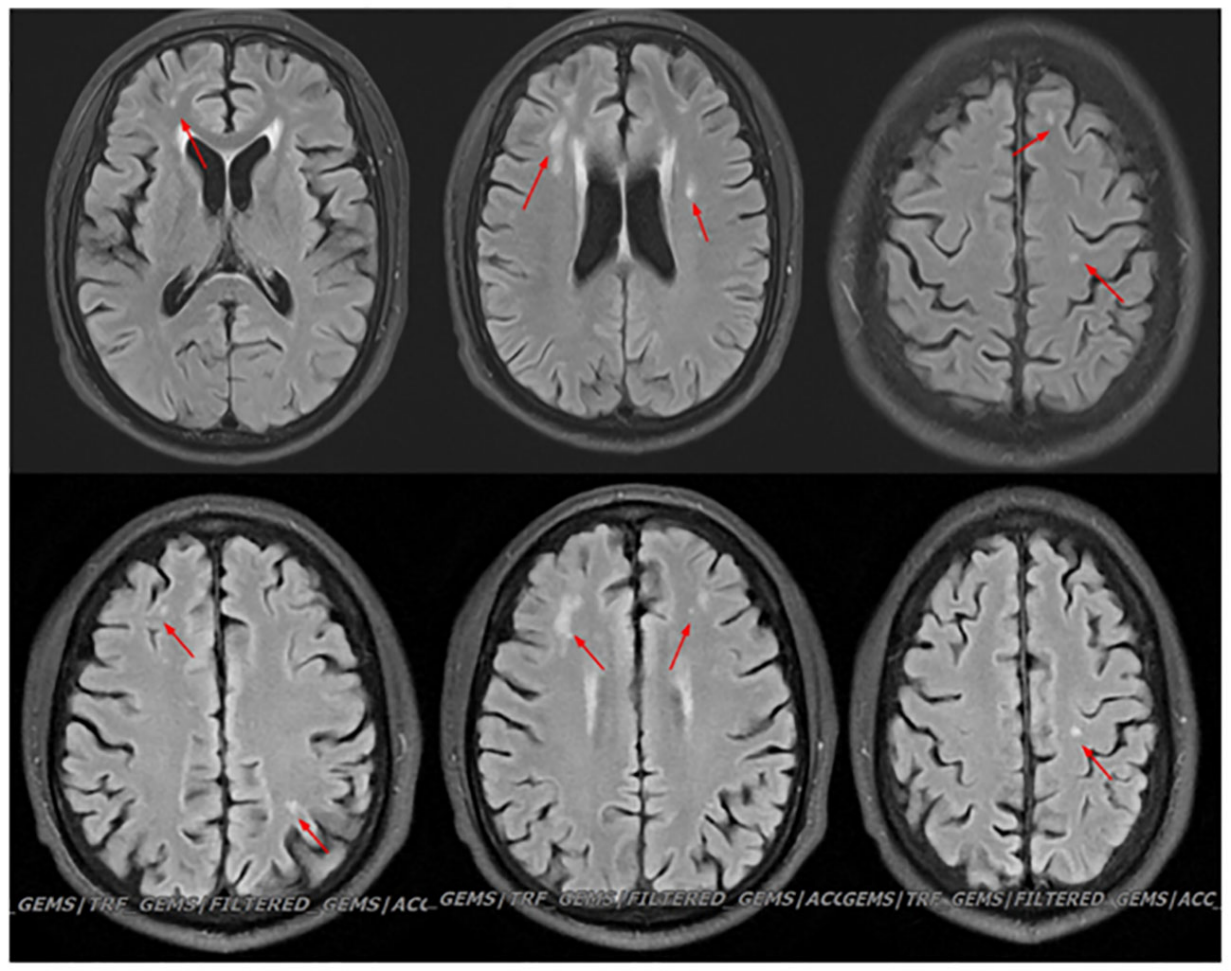
Head MRI shows: 1. Multiple abnormal signal shadows in the bilateral semioval center, bilateral lateral ventricular periphery, bilateral temporal lobes, bilateral insular cortex, and pons. There was no significant change in the result of reexamination.

The 55-year-old patient, a farmer, was admitted to the hospital due to a one-year diagnosis of brucellosis and weakness in both lower limbs for the past two weeks. One year before admission, the patient experienced fever, sweating, and headache, which slightly improved with self-administration of painkillers. One week later, the patient developed a loss of appetite, fever, and numbness in the left lower limb. The patient had a history of sheep contact. The results of the brucellosis antibody test conducted at the local hospital indicated a positive result for the brucellosis tiger red plate agglutination test (+) and a brucellosis test tube agglutination test result of 1:200 (++++) (**Figure 1B**). The patient was initially prescribed oral treatment consisting of “doxycycline + cephalosporin antibiotics + ciprofloxacin” and The patient’s medical nature was poor, and his condition had been aggravated several times since his diagnosis until he came to our hospital this time, and he was treated in different hospitals (**Figure 2**). Two weeks ago, the patient suddenly experienced weakness in both lower limbs, which gradually worsened, accompanied by numbness of the cheek and around the mouth, askew of mouth, choking cough of drinking water, incontinence of stool and urine (continuous alternation of constipation and diarrhea), decreased tactile sensation of both lower limbs, no fall and loss of unconsciousness. Upon revisiting the local hospital, the head MRI revealed multiple abnormal signals in various regions, including the bilateral semioval center, bilateral periventricular area, bilateral temporal lobe, bilateral insular cortex, and pons (**Figure 3**). Subsequently, the patient was transferred to our hospital for further medical treatment. Past medical history includes a 30-year history of Hepatitis B, liver cirrhosis, and spleen enlargement, with regular use of “Entecavir capsules”. Family history reveals that both the patient’s mother and sister passed away due to “liver cirrhosis”, the others were not special.

**Figure 4:**
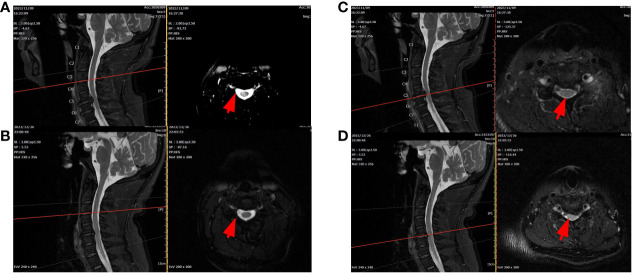
Cervical MRI **(A, C)**: T2 signals on the right side of the spinal cord were enhanced at the C3/4 level. C5/6 disc herniation with left posterior hernia and increased spinal T2 signal at corresponding levels. After treatment, C3/4 reinforcement was significantly reduced, but C5/6 did not change **(B, D)**.

Physical examination after admission: body temperature: 36.6°C, heart rate: 78 beats/min, blood pressure: 118/70mmHg. Poor articulation, slow reaction, not fluent language. The left nasolabial groove becomes shallow, the mouth Angle is skewed to the right, and the extended tongue is skewed to the right. Double upper limb muscle strength level 4, double lower limb muscle strength level 3; Increased muscle tone in left upper limb and lower limb; Bilateral biceps and triceps hyper reflexes; Both knee tendon reflexes are active; Hoffman sign (+); Bilateral finger nose test and alternate test were not stable. The heel knee shin test and Romberg sign could not be completed. Bilateral Babinski sign (+); Sensory loss was diagnosed as left T8, right T6 below, shallow sensation decreased, bilateral deep sensation disappeared; Anal sphincter dysfunction. Gait and posture: flat car thrust; The serological examination during the course of the disease is shown in the figure (**Table 1**). Brucellosis tiger red plate agglutination test: positive (+), Brucellosis test tube agglutination test positive (+); Autoantibody, Five items of thyroid function, Blood ammonia, Urine analysis + Sediment, Fecal routine were not significantly abnormal; MR Cervical vertebra + hyperlipidemia imaging plain scan (**Figure 4**): On the C3/4 section (**Figure 4A**), there was an increased T2 signal intensity in the right part of the spinal cord. C5/6 intervertebral disc bulging and protrusion, corresponding to spinal cord degeneration **Figure 4C**. The limb electromyography, visual evoked potential, and auditory evoked potential tests revealed the following findings:1.Peripheral neurogenic damage in both lower extremities with involvement of sensory and motor fibers. 2. Right median nerve damage, consistent with electrophysiological manifestations of carpal tunnel syndrome. 3. Abnormality in visual evoked potentials, suggesting suprachiasmatic damage.4. Bilateral central damage observed in brainstem auditory evoked potentials. Additionally, the electroencephalogram showed mildly abnormal results.

**Table 1:** Serological test resultsSerological test results.

	2023/11/8	2023/11/15	2023/11/26	2023/12/26
PLT(10^9/L)	52	51	53	72
RBC(10^12/L)	4.17	3.73	3.62	3.73
HGB(g/L)	141	123	123	129
WBC(10^9/L)	3.11	2.09	3.1	3.29
CRP (mg/L)	0.65	0.81	2.84	3.5
GGT(U/L)	128.8	–	–	160.3
K(mmol/L)	3.40	3.48	3.83	3.19
HP(umol/L)	23.1	–	–	63.6

The patient was diagnosed with brucellosis before admission and was immediately given an anti-infection regimen: “Ceftriaxone sodium for injection (2g bid ivgtt), Amikacin injection (0.4g bid ivgtt), Rifampicin (0.6g qd P.O), Doxycycline (100mg bid P.O)”; After consultation by a neurologist, it is suggested that the patient’s myelitis may be, following treatments were added to nourish the nerves and relieve spinal cord edema: “Give mecobalamin tablets (0.5mg tid P.O), vitamin B1 tablets(10mg tid P.O), Sodium aescin for injection (20mg qd ivgtt)”; At the same time to improve the lumbar puncture examination. Cerebrospinal fluid (CSF) results showed abnormalities (**Figure 1A**), and myelitis was diagnosed and transferred to the Department of Neurology for continued treatment. The adjustment of the medication regimen was as follows: Dexamethasone sodium phosphate injection (10mg qd ivgtt, reduced to 8mg after 1 week) was given to reduce inflammation and prevent adhesion; Mecobalamin tablets were changed to mecobalamin injection (0.5mg qd im), which helps in nerve nourishment. Although the revised treatment led to some improvement in the patient’s symptoms, the overall effect was not satisfactory. Previous studies have demonstrated the potential of high-dose hormone combined with gangliosides in promoting nerve tissue repair (Geisler et al., 1993). Therefore, “compound brain peptide ganglioside injection (4 branches qd ivgtt, stopped after 10 days)” was given. On the 9th and 16th day of admission, the cerebrospinal fluid reexamination (**Figure 1A**) revealed a decrease in the white blood cell count and protein content, the patient gradually got out of bed from the paraplegic state, the reaction ability was enhanced, the mood was improved, the stool was still not fully controlled, and the urine changed from incontinence to self-control. However, due to myelitis resulting from brucellosis, the aforementioned examinations could not be conclusively confirmed. We obtained consent from patients and their families to collect 3ml of cerebrospinal fluid via lumbar puncture during the middle and later stages of treatment at our hospital. The specimens were sent to Zhengzhou Jinyu Clinical Laboratory for pathogen detection using MetaCAP™, which confirmed our diagnosis (**Figure 1C**). Brucella sequence was detected in the cerebrospinal fluid, and its quantity significantly decreased during the late stage of treatment. Re-examination of head MRI before leaving the hospital: 1. High signal intensity in white matter (FazekasII); After requesting a treatment plan (anti-brucellosis + relief of myelitis), the patient and their family members were discharged and transferred to a local hospital for further treatment. During the hospital, the patient was informed of the relevant traumatic examination and the use of hormone therapy, explaining the necessity and risk factors, and the patient was informed and signed the informed consent.

According to our regular follow-up, the patient improved two cerebrospinal fluid examinations (**Figure 1A**) after returning to the local hospital for treatment. More than 10 days after discharge from the local hospital, the patient had no obvious cause of diarrhea, poor appetite, and weakened movement. The patient revisited our hospital’s infection department for re-examination of the cerebrospinal fluid, but no significant abnormalities were detected (**Figure 1A**). The cervical MRI showed that the T2 signal of the right part of the spinal cord at the level of C3/4 was increased, and the range was significantly smaller than before (**Figures 4A, B**). For C5/6 disc herniation, corresponding to spinal cord degeneration, no significant changes were observed (**Figures 4C, D**). The serologic brucella titer increased compared to before. Considering that diarrhea caused repeated illness, the treatment of “anti-brucellosis + anti-virus “ was given after admission, and the existing treatment was continued after discharge. During the telephone follow-up one month after leaving the hospital, the patient and his family members reported that their symptoms were better than before, their condition was stable, and they took drugs regularly.

## 3 Discuss

The occurrence, development, and prognosis of neurobrucellosis are complex, posing a challenging diagnostic problem due to the absence of a unified standard. Hence, it is crucial to gather a detailed medical history, assess clinical manifestations, conduct auxiliary examinations, consider epidemiological factors (Naderi et al., 2022), and identify potential coexisting infectious diseases (D'Anastasio et al., 2011). Haji-Abdolbagi et al. (2008) conducted studies indicating that the standard diagnosis of neurobrucellosis involves: 1) a history of epidemiological contact; 2) neurological symptoms; 3) abnormalities in cerebrospinal fluid, indicating elevated protein levels or mild to moderate lymphocytosis; glucose and chloride levels may be normal in the early stage and decrease in the later stage; 4) positive immune tests in serum, bone marrow or cerebrospinal fluid 5) Brucella standard treatment improved clinical or laboratory results; 6) ruled out other suspicious diseases. The result of an MRI scan was included as diagnostic criteria in another (Guven et al., 2013). Neuroimaging findings of brucellosis typically varied across four modes: normal, inflammation, white matter changes, and vascular changes (Jabbour and Tabbarah, 2011). Currently, laboratory detection methods for brucellosis primarily involve bacterial culture, serum immunological detection, PCR, and other techniques. The positive culture of Brucella in blood or cerebrospinal fluid serves as the “gold standard” for diagnosing NB (Shakir et al., 1987). NB cerebrospinal fluid typically exhibits elevated protein levels, increased white blood cell count (predominantly lymphocytes), and decreased levels of glucose and chloride (Zheng et al., 2018), and serological tests have a positive rate of only 28% (Guven et al., 2013), cannot differentiate between current and previous infections, and consequently tend to produce false positive results (Papadopoulos et al., 2021), thereby limiting their broad utilization. Relying solely on laboratory results is insufficient for an accurate diagnosis. Hence, there is a need for more sensitive and specific methods to aid clinicians in the early and prompt diagnosis of NB.Recently, sequencing technology has been extensively employed for microorganism identification, demonstrating remarkable sensitivity and specificity. For instance, macro gene second-generation sequencing (mNGS) and multi-PCR targeted sequencing technology (tNGS) (Gu et al., 2019), However, mNGS often leads to missed detection, and the range of tNGS pathogen spectrum is limited (Yu and Nielsen, 2010). Taking into account these limitations, we employ the macro gene capture method. MetaCAP™ is an innovative pathogen detection technique that integrates probe capture technology with NGS technology. It possesses features such as a broad spectrum, high sensitivity, coverage of the entire genome of RNA viruses, compatibility with virus homologous mutation, covering the drug resistance sites in pathogens, and high-cost performance, among others. Studies have shown that the product’s notable sensitivity and specificity in detecting sterile samples like tissue and body fluids (He et al., 2023). This is the first reported case of using cerebrospinal fluid MetaCAP™ to diagnose neurobrucellosis. NB is a rare but potentially fatal complication of brucellosis. It is essential to identify an efficient diagnostic test that can facilitate doctors to diagnose and treat patients with negative serum or cerebrospinal fluid to prevent severe outcomes (Papadopoulos et al., 2021).

In this case, the patient’s head MRI (**Figure 2**) revealed multiple abnormal signal shadows, indicating potential inflammatory demyelination associated with neurobrucellosis. Although the patient’s clinical symptoms improved significantly and cerebrospinal fluid (CSF) levels gradually decreased, there was no improvement in the white matter lesion or appearance, which was also observed in the study, the patient’s signal was enhanced at the C3/4 level, suggesting inflammatory lesions caused by Brucella. However, after treatment, the extent of the lesions significantly reduced (**Figures 4A, B**). These findings indicate the effectiveness of our treatment approach. Clinically, vestibulocochlear nerve involvement has been reported as a common and unique feature of neurobrucellosis to distinguish it from neurotuberculosis (Zheng et al., 2018) (Erdem et al., 2015). Although the patients in this study did not exhibit significant hearing or vision loss, EMG results indicated damage to the optic and auditory nerves. In addition, while elevated CSF protein levels are a known feature of neurotuberculosis, they are not commonly found in cases of neurobrucellosis. The CSF protein levels in the patients of this study were moderately elevated (Naderi et al., 2022). In addition, the possibility of immune-inflammatory lesions should also be considered, such as neuro-Behcet syndrome. However, in this patient, immune-related indicators were negative, there was no evidence of multiple organ damage, and autoimmune myelitis was not supported by any evidence.

The treatment principle of neurobrucellosis involves long-term, regular, and combined treatment, as it cannot be cured by a single drug treatment and is prone to relapse. The selection of antibiotics with strong intracellular and central nervous system penetration is crucial (Erdem et al., 2016). In China, the current standard therapeutic drugs include doxycycline and rifampicin. These drugs are combined with an aminoglycoside, ceftriaxone, or one of the quinolone antibiotics for a treatment duration of 3-6 months (Erdem et al., 2016). Corticosteroids protect spinal cord function by inhibiting neuroinflammation related to spinal cord injury and preventing a systemic immune response (Chio et al., 2021). Studies (Wang et al., 2020) have demonstrated that the utilization of corticosteroids can elevate the likelihood of experiencing adverse drug reactions. Ganglioside (GM) predominantly resides within the brain tissues of all mammals and serves as a structural constituent of human nerve cell membranes (Yuki, 2001). Its involvement in the body encompasses regulatory functions in development, differentiation, and pathological alterations, ultimately safeguarding nerve cell functionality and fostering nerve regeneration (Tandon et al., 2019). Studies (Wang et al., 2018)have shown that the combination of high-dose methylprednisolone pulse therapy and gangliosides confers complementary benefits, amplifies the anti-nerve cell injury effect, and augments clinical treatment outcomes. In our case, the effect of dexamethasone sodium phosphate alone was not obvious, but the effect was significant after the combination of hormone and compound brain peptide ganglioside. Acupuncture combined with rehabilitation exercises effectively treats skeletal muscle atrophy in patients (Shou et al., 2021). The patient started exercising early in the treatment and got out of bed slowly with the help of family members during the middle of the treatment. Although the patient had some difficulty maintaining balance and was prone to falling, it was a significant improvement from their previous paraplegic state. NB patients may develop behavioral and neuropsychiatric disorders, such as depression (He et al., 2022). When history was taken, patients reported low mood, slowness, and non-response to questions, and significant cognitive and mood improvements were observed only after treatment without any antidepressant or antipsychotic therapy, which is consistent with previous studies (Eren et al., 2006). Brucella is an intracellular bacterium that can enter a dormant state and reactivate after a variable clinical incubation period. In this case, we consider that the sudden worsening of the patient’s condition could be attributed to the activation of Brucella and irregular medication.

In conclusion, considering that brucellosis affects many individuals, clinicians in epidemic areas and neurology departments must comprehensively understand the clinical manifestations and diagnostic criteria for brucellosis involving the central nervous system. Consequently, we report a case study of a patient who exhibited persistent myelitis symptoms throughout the disease, and these symptoms showed gradual improvement with appropriate treatment. Furthermore, to the best of our knowledge, this is the first reported instance of utilizing MetaCAP™ for diagnosis, which holds significant importance in diagnosing patients with neurological symptoms and potentially reducing mortality and long-term complications. We discussed the diagnosis, treatment, and potential correlation between these two conditions in detail, thereby contributing to the existing literature gap on neurobrucellosis in myelitis.

## Data availability statement

The original contributions presented in the study are included in the article/**Supplementary Materials**, further inquiries can be directed to the corresponding author/s.

## Ethics statement

The studies involving humans were approved by The Ethics Committee of the First Hospital of Lanzhou University. The studies were conducted in accordance with the local legislation and institutional requirements. The participants provided their written informed consent to participate in this study. Written informed consent was obtained from the individual(s) for the publication of any potentially identifiable images or data included in this article. Written informed consent was obtained from the participant/patient(s) for the publication of this case report.

## Author contributions

XYM: Data curation, Methodology, Project administration, Writing – original draft, Writing – review & editing. YW: Data curation, Writing – review & editing. QOW: Data curation, Writing – review & editing. XMM: Data curation, Writing – review & editing. QAW: Resources, Writing – review & editing, Data curation. QG: Resources, Writing – review & editing, Funding acquisition, Supervision.
