# Supplementary material for: Brucellosis infection complicated with myelitis: a case report and literature review
Source: Front Cell Infect Microbiol. 2024 May 16;14:1378331. doi: 10.3389/fcimb.2024.1378331 (PMC11137236; doi:10.3389/fcimb.2024.1378331)
Supplement: Supplementary file 3 [file DataSheet_1.pdf]

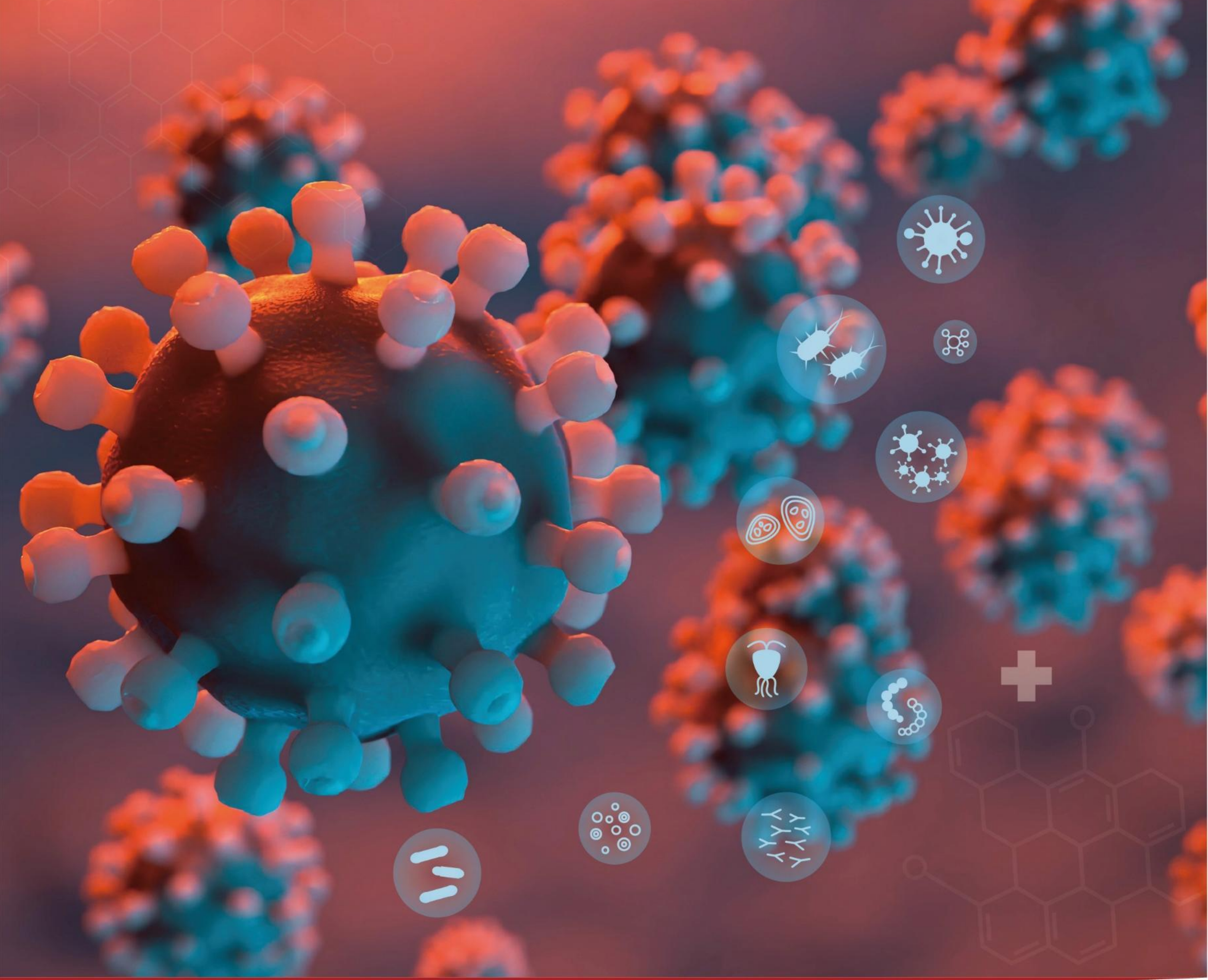

MetaCAP™ for Pathogenic Detection Report

Name	Gadong Ma	Gender	Male	Age	55 years old
Hospital	The First Hospital of Lanzhou University	Department	–		
Specimen	CSF（In the treatment）	Barcode	2805394706		
Report Date	2023-11-25				

Information of Sample

Name	Gadong Ma	Gender	Male	Age	55 years old
Hospital	The First Hospital of Lanzhou University			Department	–
Physician	–	In–Patient ID	–	Bed number	–
Specimen	CSF（In the treatment）	Barcode	2805394706	Lab code.	MC23T0395
Collected Data	2023–11–17	Received Data	2023–11–23	Report Date	2023–11–25
Inspection items	High–throughput sequencing of pathogenic microbial nucleic acids	Inspection method	High–throughput sequencing		

1. Specific Microbes
（Mycobacterium, Mycoplasma, Chlamydia, Rickettsia, Spirochaetales）

Genera				Species/Complex		
Type	Name	Reads	Relative abundance	Name	Reads	Coverage
G–	*Brucella*	1534	82.07%	*Brucella melitensis*	1513	90513/3312779 (2.73%)

2. Bacteria

Genera				Species/Complex		
Type	Name	Reads	Relative abundance	Name	Reads	Coverage
Undetected						

3. Fungi

Genera				Species/Complex		
Type	Name	Reads	Relative abundance	Name	Reads	Coverage
Undetected						

4. DNA/RNA Viruses						
Genera				Species/subtypes		
Type	Name	Reads	Relative abundance	Name	Reads	Coverage
dsDNA	*Lymphocryptovirus*	825	98.64%	*Epstein–Barr virus*	825	31083/172204 (18.05%)

5.Parasites						
Genera				Species		
Type	Name	Reads	Relative abundance	Name	Reads	Coverage
Undetected						

6. Suspected Human Microbiome

A diverse microbial flora is found in all areas of the human body exposed to the environment such as skin, mouth, nose, ears, eyes, urinary track and the full gastrointestinal tract. Although most bacteria in the normal flora are harmless in healthy individuals, these microbes frequently cause disease in immunocompromised patients.

Genera			Species/Complex	
Type	Name	Reads	Name	Reads
G+	*Cutibacterium*	83	*Cutibacterium acnes*	82
G+	*Staphylococcus*	37	*Staphylococcus epidermidis*	21

Reference

1.Michael M J , Binnicker M J , Sheldon C , et al. A Guide to Utilization of the Microbiology Laboratory for Diagnosis of Infectious Diseases: 2018 Update by the Infectious Diseases Society of America and the American Society for Microbiology [J]. Clinical Infectious Diseases, 2018, 67(6):813-816.

2.Chiu C Y , Miller S A . Clinical metagenomics [J]. Nature Reviews Genetics, 2019, 20(6): 341-355.

3.Byrd A L , Belkaid Y , Segre J A . The human skin microbiome [J]. Nature Reviews Microbiology, 2018, 16(3).

4.He T , Kaplan S , Kamboj M , et al. Laboratory Diagnosis of Central Nervous System Infection [J]. Current Infectious Disease Reports, 2016, 18(11):35.

5.Miller S , Naccache S N , Samayoa E , et al. Laboratory Validation of a Clinical Metagenomic Sequencing Assay for Pathogen Detection in Cerebrospinal Fluid [J]. Genome Research, 2019, 29(5): 831-842.

6.Huffnagle G B , Dickson R P , Lukacs N W . The Respiratory Tract Microbiome and Lung Inflammation: A Two-way Street [J]. Mucosal Immunology, 2017, 10(2):299-306.

7.Mamanova, L., Coffey, A., Scott, C. et al. Target-enrichment strategies for next-generation sequencing. Nat Methods 7, 111 – 118 (2010).

This detection is only responsible for the samples received. If you have any doubts about the results, please contact us within 7 days after the report is released. Thank you for your cooperation!

	Technologist	Bei Xu	Approver	Mengfei Zhang
	Lab： Zhengzhou KingMed Diagnostics, Inc	Web: www.kingmed.com.cn<br>Customer ServiceI：4001-111-120		
	Add： Building 1#, Binhe No.1 City, 200 meters east of 15th Street and Hongyun Road, Zhengzhou Economic and Technological Development Zone, Zhengzhou, China			
